# The Role of AIM2 in Cancer Development: Inflammasomes and Beyond

**DOI:** 10.7150/jca.101473

**Published:** 2025-01-01

**Authors:** Lina Sui, Yuling Xi, Siping Zheng, Qiuxiang Xiao, Zhiping Liu

**Affiliations:** 1 Center for Immunology, Key Laboratory of Prevention and Treatment of Cardiovascular and Cerebrovascular Diseases, Ministry of Education, Gannan Medical University, Ganzhou, Jiangxi 341000, China.; 2 School of Basic Medicine, Gannan Medical University, Ganzhou, Jiangxi 341000, China.; 3 Department of Pathology, The First-Affilliated Hospital, Gannan Medical University, Ganzhou, Jiangxi 341000, China.

**Keywords:** AIM2, Inflammasome, Cancer, AKT

## Abstract

Absence in melanoma 2 (AIM2) protein functions as a double-stranded DNA sensor and is critical for host defense against intracellular bacterial and viral pathogens. Recent research has highlighted the significance of AIM2 in the pathogenesis of diverse malignancies. Through its recognition of foreign or intracellular dsDNA, AIM2 triggers inflammasome activation, resulting in the release of pro-inflammatory cytokines such as IL-1β, IL-18, and induction of pyroptosis. Additionally, AIM2 can engage alternative signaling pathways, such as AKT and NF-κB, independent of inflammasome activation, to modulate cancer progression. This review provides a comprehensive overview of recent advancements in understanding the involvement of AIM2 in the pathogenesis of different types of cancer through both inflammasome-dependent and inflammasome-independent mechanisms. Furthermore, we discuss the potential applications and challenges associated with targeting AIM2 in cancer therapy.

## Introduction

Absent in melanoma 2 (AIM2) was discovered in melanoma in 1997 and was initially characterized as a tumor suppressor gene. Research has shown that overexpression of AIM2 can potentially reverse the malignant characteristics of melanoma cells^1^. AIM2 is notable as the first non-NLR member capable of forming an inflammasome^2^. AIM2 contains a C-terminal HIN-200 domain and an N-terminal Pyrin domain (PYD)^3^. The HIN domain is directly responsible for binding to double-stranded DNA (dsDNA) in the cytoplasm. The HIN-200 and the Pyrin domain form an intramolecular complex and remain in the autoinhibited state. The AIM2 inflammasome has been found to play a significant role in the host's immune response to bacterial and viral infections. In the context of infection, DNA originating from either a pathogen or compromised host cells is discharged into the cytoplasm, where it is detected by cytoplasmic DNA sensors. For example, dsDNA in the cytoplasm induces activation of AIM2, leading to direct binding and interaction of PYD of AIM2 with ASC. In turn, the CARD of ASC binds to the CARD of pro-caspase-1, promoting caspase-1 activation and maturation of the downstream inflammatory cytokines IL-1β and IL-18^4^.

Endogenous and exogenous dsDNA, with a minimum length of 80 base pairs, are capable of binding to the C-terminal HIN-200 domain of AIM2. Subsequently, the PYD domain is released from the intramolecular complex and interacts with the ASC^5^, ^6^. The CARD domain of ASC then binds to the CARD domain of pro-caspase-1, forming a macromolecular complex that fulfills the essential structural requirements of the inflammasome^3^. AIM2 is expressed in the cytoplasm and recognizes dsDNA in a manner that is not dependent on specific sequences. The sugar-phosphate backbone of dsDNA interacts with the positively charged HIN-200 domain of AIM2 through electrostatic attraction. The activation of the AIM2 inflammasome through PYD self-oligomerization leads to the activation of caspase-1, which in turn cleaves pro-IL-1β and pro-IL-18, resulting in the release of mature IL-1β and IL-18 from the cells.^7^, ^8^. Additionally, caspase-1 cleaves the N-terminal fragment of gasdermin D (GSDMD), inducing pyroptosis, a form of cell death^3^, ^9^, ^10^.

The inflammasome is a large molecular complex formed by cytoplasmic pattern recognition receptors (PRRs), the adaptor protein ASC, and zymogen procaspase-1, which are integral components of the inflammatory immune response. Key sensors of the inflammasome include Nod-like receptors (NLRs), AIM2, and Pyrin^11^. The assembly of the inflammasome complex activates caspase-1 through autoproteolytic hydrolysis. Subsequently, active caspase-1 processes the precursor cytokines IL-1β and IL-18 into their mature, active forms. Additionally, active caspase-1 induces a form of inflammatory cell death known as pyroptosis by cleaving GSDMD. The cleavage of GSDMD releases its N-terminal domain, which forms pores in the plasma membrane, resulting in osmotic imbalance, cell swelling, loss of membrane integrity, and eventual cell rupture^4^, ^12^. Recent research has highlighted the significant role of the inflammasome in cancer, impacting various aspects such as inflammation, cell growth and death, blood vessel formation, and metastasis. In specific contexts, inflammasomes demonstrate varying effects on cancer development^13^. They may either initiate carcinogenesis and sustain the malignancy microenvironment, or alternatively, exhibit anticancer properties by modulating immune responses^14^.

AIM2 has the capacity to impede tumor progression through the facilitation of apoptosis, suppression of cell proliferation, and initiation of autophagy^15^-^17^. Conversely, in select cancers or particular stages of cancer, AIM2 may stimulate the proliferation of cancer cells^18^, ^19^. Furthermore, AIM2 has the potential to impact cancer development through either an inflammasome-dependent or inflammasome-independent mechanism. This review primarily focuses on recent research regarding the diverse functions of AIM2 independent of the inflammasome in inflammatory responses, cell proliferation, apoptosis, and metastasis across various cancer types.

### AIM2 acts in tumors dependent on inflammasome signaling

AIM2 functions as a dsDNA sensor that initiates inflammasome signaling in various cancer types. Previous research conducted by our team revealed a notable decrease in AIM2 expression within colorectal cancer (CRC) tissues compared to control samples, correlating with downstream inflammasome molecules ASC and IL-18^20^. In renal cell carcinoma (RCC), a separate investigation demonstrated that a conditionally replicating adenovirus containing AIM2 effectively suppressed cell proliferation, induced apoptosis, and hindered lung metastasis by activating the inflammasome pathway^21^. Furthermore, AIM2 has been demonstrated to facilitate the transition of tumor-associated macrophages (TAMs) from an anti-inflammatory M2-type to a pro-inflammatory M1-type by activating inflammasome signaling, thereby promoting tumor rejection and impeding tumor cell proliferation^16^. In the context of hepatocellular carcinoma (HCC), AIM2 has been found to suppress HCC cell proliferation, colony formation, and invasion by inducing pyroptosis through inflammasome formation, consequently inhibiting the mTOR-S6K1 pathway and hindering tumor progression^22^. However, another study discovered that AIM2 promotes HCC by activating the inflammasome, leading to increased expression of IL-1β and IL-18, and amplifying the inflammatory response^23^. In the context of cervical cancer, the AIM2 inflammasome inhibits human papillomavirus (HPV)-infected cervical cancer by inducing cellular pyroptosis through extracellular vesicles^24^. Additionally, activation of the AIM2 inflammasome suppresses bladder cancer (BLCA) and improves the efficacy of BCG vaccine therapy in BLCA^25^. Research has shown that activation of the AIM2 inflammasome in nasopharyngeal carcinoma leads to increased secretion of tumor-derived IL-1β, resulting in the recruitment of a significant population of tumor-associated neutrophils (TANs) and suppression of tumor growth in murine models^26^.

Interestingly, in prostate cancer, hypoxia has been found to stimulate the transcriptional activity of NF-κB in human normal prostate epithelial cells and THP-1 cells, a human monocyte cell line. This activation subsequently triggers AIM2 inflammasome, leading to the promotion of chronic inflammation within the prostate gland and the progression of prostate cancer^27^. However, the mechanism by which activation of the AIM2 inflammasome contributes to the progression of prostate cancer remains unclear. In lung adenocarcinoma (LUAD), AIM2 has been shown to induce the transcriptional regulation of NF-κB and STAT1 via inflammasome activation, leading to the promotion of epithelial-mesenchymal transition (EMT) processes and increased PD-L1 expression. Additionally, AIM2 may facilitate LUAD development by influencing cell cycle progression, specifically by causing cell accumulation in the G2/M phase and inhibiting cell proliferation, as well as by activating calpain and IL-1α or modulating mitochondrial dynamics^18^, ^28^-^30^.

**Figure 1:**
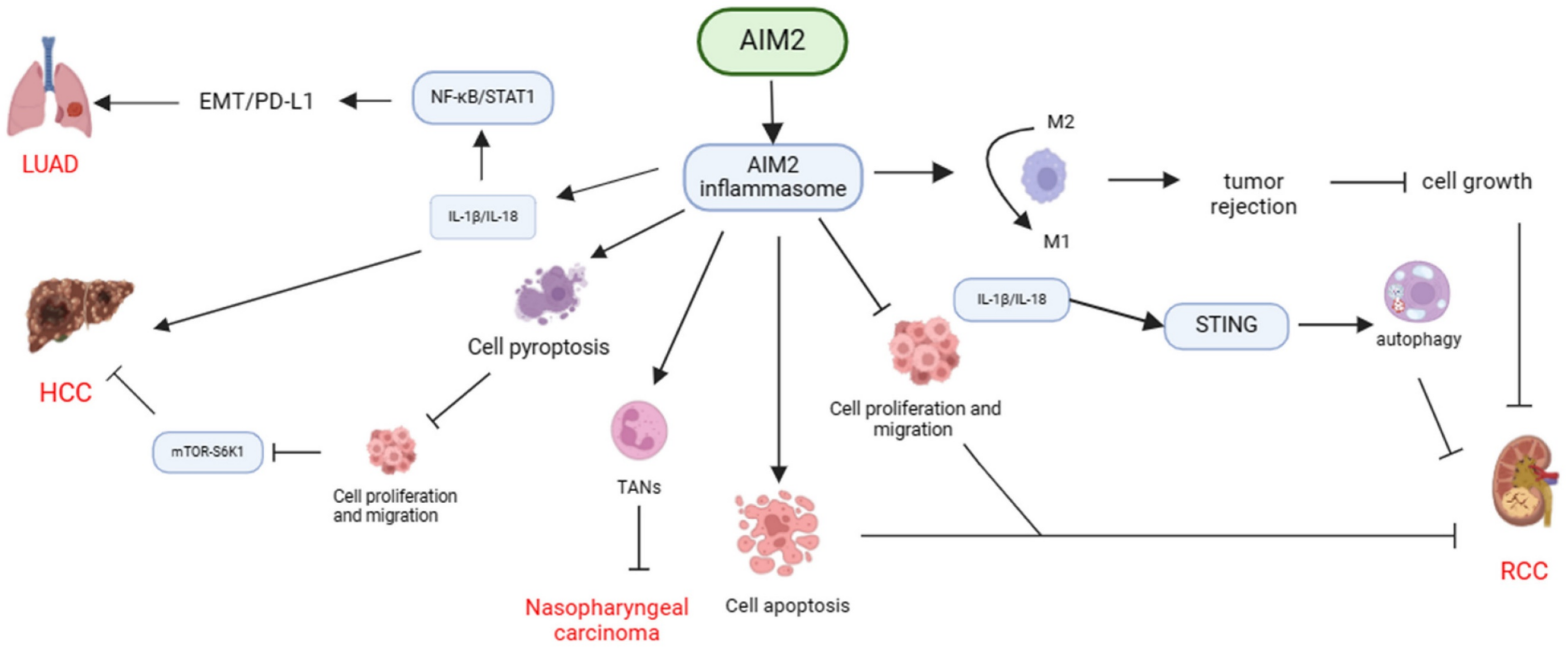
** Mechanisms of AIM2 regulation in various cancers through dependence on inflammasome signaling.** AIM2 exhibits diverse functions in different types of cancers through its dependence on inflammasome signaling. It can suppress cancer cell proliferation, induce apoptosis or pyroptosis, enhance tumor rejection by transforming TAMs, and impede the progression of RCC, nasopharyngeal carcinoma, and HCC by recruiting TANs. Conversely, AIM2 can also facilitate the development of HCC and LUAD by activating inflammasome signaling, promoting EMT, or influencing the cell cycle.

In conclusion, AIM2 exhibits diverse functions in the pathogenesis of various cancers through its interaction with inflammasome signaling pathways. AIM2 has been shown to exert anti-tumor effects by inhibiting cancer cell proliferation, inducing apoptosis, pyroptosis, and enhancing the immune response against cancer cells by modulating TAMs and TANs. Conversely, AIM2 can also facilitate cancer progression by promoting EMT and affecting the cell cycle, highlighting the complex role of AIM2 in cancer development (**Table 1, Figure 1**).

### AIM2 acts in tumors independently of inflammasome signaling

#### CRC

CRC is a prevalent malignancy within the gastrointestinal tract, with incidence and mortality rates ranking third and second, respectively^31^. Diminished AIM2 expression has been noted in CRC tissues^32^-^36^. Through a combination of bioinformatics analysis and examination of clinical tissue samples, it was determined that AIM2 expression levels were notably lower in tumor tissue samples from CRC patients compared to those from healthy individuals^32^. Several other studies have reported comparable findings, indicating that AIM2 expression is notably decreased in primary CRC tissues compared to adjacent normal colorectal tissues. Furthermore, AIM2 expression was found to be diminished or absent in over three-quarters of tumor tissues in comparison to epithelial cells in normal tissues^33^. Additionally, reduced AIM2 expression was linked to the depth of CRC infiltration, TNM stage, and lymph node metastasis^32^. Moreover, the expression of AIM2 and gene mutations in CRC tissues were found to be positively associated with the overall survival rate of CRC patients^33^-^36^.

A recent study demonstrated that the transfection of AIM2 into HCT116 cells did not result in the activation of caspase-1 or the cleavage of IL-1β precursor, indicating that AIM2 transfection did not activate the inflammasome in CRC cells. Instead, AIM2 was found to induce the expression of HLA-DRA and HLA-DRB by modulating CIITA expression, and inhibit CRC cell proliferation through the IFN/AIM2/ISG cascade^37^. Another study discovered that AIM2 suppressed CRC cell viability by inhibiting the PI3K/AKT pathway and caused an increase in the percentage of cells in G1 and G2/M phases, without affecting the S phase, ultimately promoting cell apoptosis^38^. Additionally, AIM2 impeded the cell cycle progression from G2 to M phase, consequently impeding proliferation and suppressing the growth and invasion of CRC cell lines, albeit without inducing apoptosis^39^. AIM2 was demonstrated to impede the proliferation and migration of CRC cells through the suppression of the AKT/mTOR signaling pathway and the inhibition of Glil expression, thereby exerting a tumor suppressor effect^17^.

The suppression of CRC by AIM2 is linked to its role in pathological development. Studies on *Aim2^-/-^* mice have shown a higher incidence of colitis-associated CRC compared to *Asc^-/-^* mice with defective inflammasomes, suggesting that AIM2's impact on CRC is not dependent on inflammasome activation. Additionally, *Aim2^-/-^*/*Apc^Min/+^* mice exhibited increased tumor burdens compared to *Apc^Min/+^* mice. Mechanistically, AIM2 interacts with and restricts the activation of DNA-dependent protein kinase (DNA-PK), thereby inhibiting Akt activation and ultimately reducing tumor burden in the CRC model^40^.

A previous study indicated that in colitis-associated CRC,there was a notable increase in both the quantity and size of tumors in the mesocolon and distal colons of *Aim2^-/-^* mice compared to WT mice. However, similar levels of activition of inflammasome or inflammation-associated molecules, such as caspase-1, IL-1β, IL-18, IL-6, TNF, and G-CSF, were observed in the colons of WT mice and *Aim2^-/-^* mice both before and after treatment. These findings suggest that AIM2 regulates tumor development through a mechanism that is independent of inflammasome activation and inflammation^41^. Subsequent research indicated that *Aim2^-/-^* mice exhibited increased cell proliferating, elevated levels of proliferation-related molecules (S100A9, SNRPD1, and DBF4, among others), heightened phosphorylation of AKT and PTEN, and upregulation of oncogenes compared to WT mice. Additionally, *Aim2^-/-^* mice demonstrated accelerated growth of colon stem cells, enhanced stem cell activity in Prom1^+^ cells following abnormal Wnt activation, and a greater propensity for tumor formation. These findings suggest that AIM2 plays a role in regulating the expansion of intestinal stem cells and serves to protect the host from CRC development^41^.

**Figure 2:**
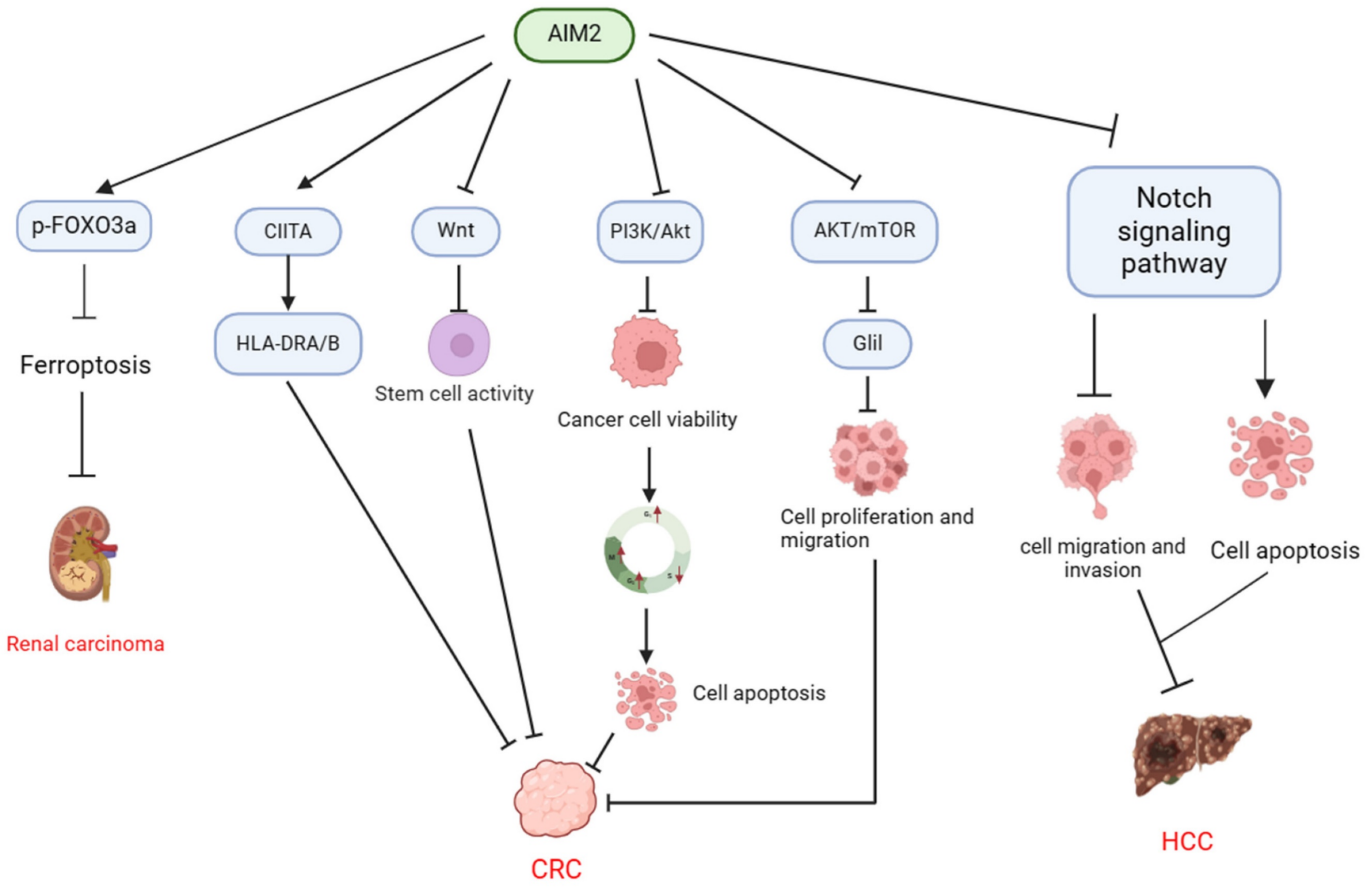
** Mechanisms of AIM2 regulation in CRC, HCC, and RCC in inflammasome-independent manner.** In CRC, AIM2 demonstrates the ability to modulate tumor stem cell activity through the regulation of CIITA expression, resulting in the induction of HLA-DRA/HLA-DRB and suppression of the Wnt signaling pathway. Additionally, AIM2 exerts an inhibitory effect on CRC cell viability by targeting the PI3K/AKT pathway, leading to cell cycle arrest at the G1 and G2/M phases and a decrease in the S phase population, ultimately promoting apoptosis. Furthermore, AIM2 exhibits a suppressive impact on CRC progression by inhibiting the AKT/mTOR and Glil pathways, consequently diminishing the proliferation and migration of CRC cells. In HCC, AIM2 facilitates cancer cell apoptosis through the suppression of the Notch signaling pathway, resulting in decreased cancer cell migration and invasion, ultimately impeding the progression of HCC. Moreover, AIM2 promotes the phosphorylation and proteasomal degradation of FOXO3a, thereby inhibiting ferroptosis and promoting the progression of RCC.

In summary, AIM2 has the ability to suppress cell proliferation through modulation of molecular expression, including S100A9, SNRPD1, and DBF4, as well as by inhibiting Akt activation to induce apoptosis. Additionally, AIM2 impacts the cell cycle by regulating stem cell activity via the Wnt signaling pathway, thereby providing protection against CRC development (**Table 2, Figure 2**).

#### Breast cancer (BC)

Based on recent global cancer data, BC has now surpassed lung cancer as the most prevalent cancer wordwild, with the highest mortality rate among female malignant tumors^42^. The expression of AIM2 has been shown to increase the number of sub-G1 phase cells and induce apoptosis in tumor cells, effectively inhibiting BC cell proliferation, and growth *in vitro,* as well as suppressing breast tumor formation *in vivo*. Additionally, AIM2 expression has been found to inhibit NF-κB transcriptional activity and desensitize TNF-α-mediated NF-κB activation, ultimately leading to pro-apoptotic outcomes^43^. Furthermore, a separate study demonstrated that heightened AIM2 expression facilitated apoptosis in BC cells via a mitochondrial pathway. This was achieved through the downregulation of the anti-apoptotic protein Bcl-xL, upregulation of the apoptotic proteins Bad and Bax, and activation of PARP^44^. Additionally, AIM2 was shown to upregulate the expression of Caspase3 and DFNA5, leading to BC cell death and ultimately inhibiting tumorigenesis^45^.

**Figure 3:**
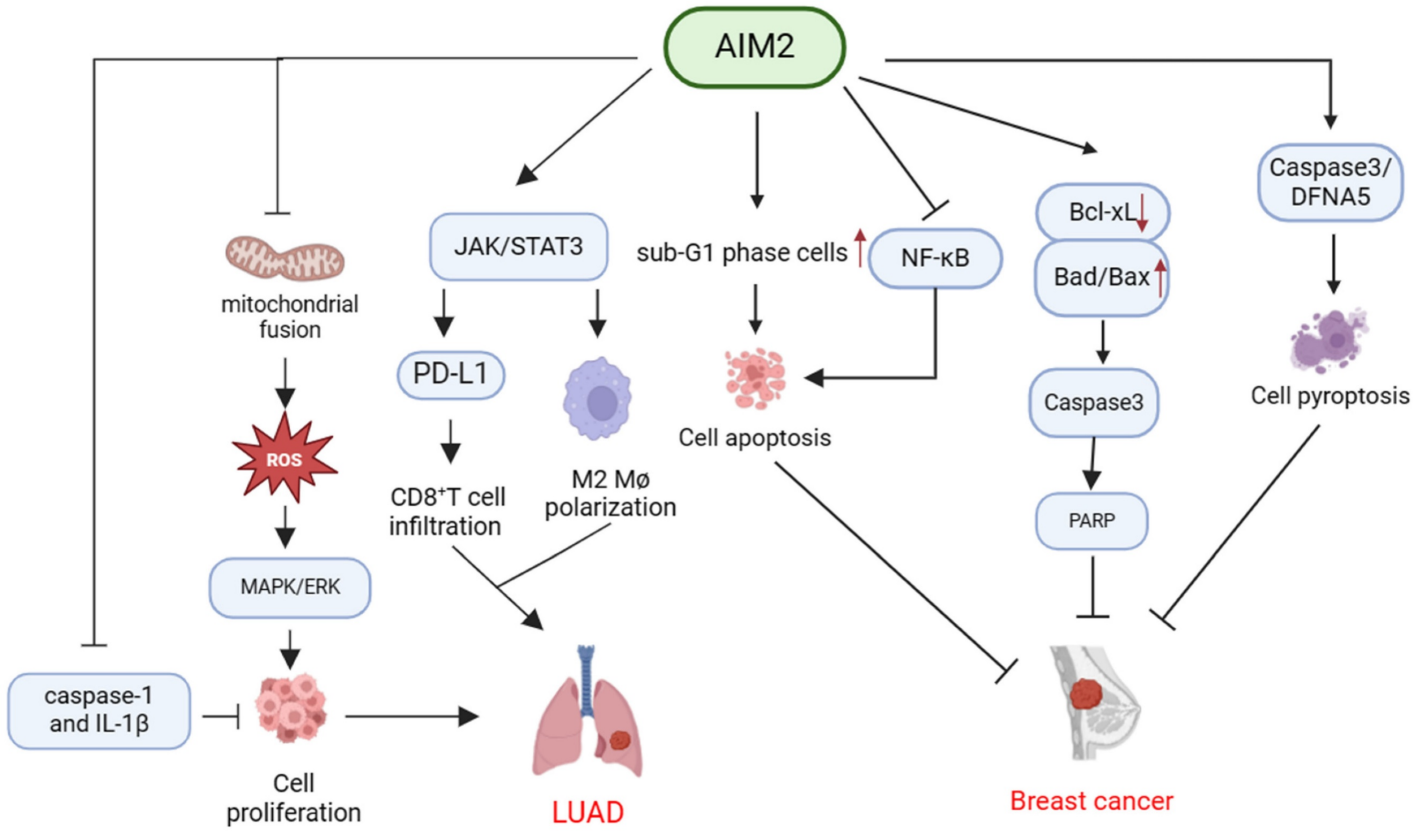
** Mechanisms of AIM2 in regulating the development of breast cancer and LUAD independent of inflammasome.** AIM2 exerts its anti-cancer effects through multiple mechanisms, including the promotion of cancer cell apoptosis via modulation of sub-G1 phase cells and inhibition of NF-κB. Additionally, AIM2 can impede cancer progression by downregulating Bcl-xL expression and upregulating Bad and Bax expression, resulting in Caspase 3 activation and subsequent PARP cleavage. Furthermore, AIM2 can enhance Caspase 3 and DFNA5 expression, leading to breast cancer cell pyroptosis and inhibition of tumor growth. Moreover, AIM2 activates the MAPK/ERK signaling pathway by inhibiting mitochondrial fusion, thereby increasing ROS production. Furthermore, AIM2 has the capability to activate the JAK/STAT3 pathway, leading to the promotion of M2 polarization in macrophages and increased PD-L1 expression, while also inhibiting CD8^+^ T cell infiltration via the PD-1/PD-L1 axis. Additionally, AIM2 can facilitate the progression of LUAD by suppressing the expression of caspase-1 and IL-1β.

In summary, AIM2 demonstrates potential anti-tumor properties in BC through the inhibition of cell proliferation and the facilitation of apoptosis or pyroptosis (**Table 2, Figure 3**).

#### Renal carcinoma (RCC)

RCC is the predominant form of urogenital cancer, exhibiting a mortality rate ranging from 30% to 40%^46^. Research has indicated that AIM2 exerts a protective function in RCC. Specifically, a study revealed that AIM2 expression was predominantly low (negative and weak) in approximately two-thirds of the 298 RCC biopsy specimens, while high (moderate and strong) expression was observed in the remaining one-third of biopsy samples^47^. Decreased AIM2 expression was found to be correlated with unfavorable prognostic outcomes, including lymph node metastasis and reduced 5-year overall and disease-specific survival rates in individuals diagnosed with RCC. Additionally, upregulation of AIM2 was observed to suppress cell proliferation, migration, and invasion, while promoting autophagy, ultimately impeding the malignant characteristics of RCC cells^47^.

A bioinformatics analysis revealed that AIM2 is prominently expressed as a regulator of pyroptosis in clear cell renal cell carcinoma (CCRCC) and is closely linked to unfavorable prognoses in CCRCC patients^48^, ^49^. Notably, AIM2 expression was notably elevated in RCC tumor tissues compared to normal tissues, leading to the promotion of RCC development through the phosphorylation and proteasomal degradation of FOXO3a. This process ultimately hinders the transcriptional effect on ACSL4, inhibits ferroptosis, and facilitates RCC progression^50^. In summary, AIM2 facilitate the progression of renal cancer through the regulation of iron-mediated cell death (**Table 2, Figure 2**).

#### Hepatocellular carcinoma (HCC)

HCC arises from hepatocytes and represents the majority of primary liver cancer cases, accounting for 90% of cases. HCC is ranked as the sixth most prevalent cancer globally in 2020 and is the third leading cause of cancer-related mortality^51^. Research has indicated a notable decrease in AIM2 expression in HCC compared to normal tissues; however, the level of AIM2 expression does not appear to correlate with recurrence-free survival or overall survival rates among HCC patients^52^. Two additional studies have corroborated the significant reduction of AIM2 expression in HCC tissues^53^, ^54^. Furthermore, diminished AIM2 expression has been strongly linked to elevated serum alpha-fetoprotein (AFP) levels, vascular infiltration, poor tumor differentiation, incomplete tumor encapsulation, and decreased postoperative survival. Additionally, AIM2 deletion has been shown to facilitate EMT activation and HCC metastasis^53^. Research has been demonstrated that the overexpression of AIM2 lead to the promotion of apoptosis and the suppression of migration and invasion in cancer cells by inhibiting the Notch signaling pathway. Additionally, AIM2 has been shown to delay tumor progression in homograft experiments involving nude mice, ultimately inhibiting the growth and metastasis of HCC^54^.

Therefore, AIM2 has the ability to suppress HCC growth and metastasis via the Notch signaling pathway without reliance on inflammasome signaling, and additionally demonstrates a protective role by inhibiting EMT (**Table 2, Figure 2**).

#### Lung adenocarcinoma (LUAD)

LUAD is a prevalent form of cancer globally and is the primary cause of cancer-related mortality^55^. Research has shown that AIM2 is significantly upregulated in the tumor tissues of patients with LUAD and is associated with a negative prognosis^56^, ^57^. A study utlizing xenograft assays and manipulating AIM2 expression in tumor cells *in vitro* demonstrated that AIM2 facilitates immune evasion in LUAD by inducing M2 macrophage polarization and PD-L1 expression via the JAK/STAT3 pathway, while also inhibiting CD8^+^ T cell infiltration through the PD-1/PD-L1 axis^57^. AIM2 has been shown to enhance cancer cell proliferation through the regulation of mitochondrial dynamics, leading to reduced mitochondrial fusion and subsequent elevation of cellular reactive oxygen species (ROS) production and activation of the MAPK/ERK signaling pathway^58^. Additionally, a recent study demonstrated that in a LAC mouse model, AIM2-deficient mice were able to mitigate KRAS-driven LUAD by facilitating the release of mature caspase-1 and IL-1β, thereby inhibiting the growth of lung cancer cells in an inflammasome adapter-independent manner^59^.

In summary, AIM2 has been shown to suppress the expression of caspase-1 and IL-1β through inflammasome-independent signaling as well as impede CD8^+^ T-cell infiltration through the JAK/STAT3 pathway, thereby facilitating immune evasion by tumors. Furthermore, AIM2 has been implicated in the promotion of lung cancer progression by modulating mitochondrial dynamics and other cellular pathways (**Table 2, Figure 3**).

#### Squamous cell carcinoma (SCC)

SCC is the second most prevalent form of cutaneous malignancy affecting the anterior region of the skull base, characterized by local infiltration of neighboring anatomical structures. The nasal cavity and paranasal sinuses are the most frequent primary sites of origin, with less common occurrences in the skin and orbit, underscoring the importance of early detection to prevent metastasis^60^, ^61^. Elevated levels of AIM2 expression have been observed in cutaneous SCC compared to normal skin, and the down-regulation of AIM2 expression has been shown to diminish cell viability and invasion while increasing apoptosis in SCC cells. This phenomenon ultimately results in the suppression of growth and vascularization of xenografts *in vivo*^62^.

AIM2 is upregulated in oral squamous cell carcinoma (OSCC). In the absence of p53, the concurrent expression of AIM2 and IFI16 can enhance cell proliferation by activating the NF-ĸB signaling pathway. Nevertheless, caspase-1 remains inactive in OSCC cells due to the inability of dsDNA to induce the formation of the AIM2 inflammasome in OSCC cells^63^. Additionally, a separate study demonstrated that AIM2 contributes to radiation resistance, migration, and PD-L1 expression in OSCC cells by activating the STAT1/NF-κB signaling pathway^64^. A study demonstrated that overexpression of AIM2 played a significant role in the tumorigenesis of OSCC, leading to increased cancer cell migration, enhanced invasive capabilities, and promotion of EMT. Futhermore, *in vivo* experiments involving the transfer of AIM2-overexpressing cancer cells into immunodeficient mice revealed accelerated tumor growth in the tongue and reduced survival rates among the mice^65^.

In contrast, the role of AIM2 in hypopharyngeal squamous cell carcinoma (HSCC) differs significantly. AIM2 expression was notably decreased in comparison to adjacent normal hypopharyngeal tissues. Reduced AIM2 expression was strongly linked to lymph node metastasis, intravascular tumor thrombosis, diminished survival rates, and unfavorable prognosis. Conversely, the expression of p-STAT3 protein and the p-STAT3/STAT3 ratio were elevated in HSCC tissues and were correlated with survival outcomes. Nevertheless, the precise mechanism through which AIM2 modulates the expression of p-STAT3 and subsequently inhibits HSCC remains unknown^66^ (**Table 2**).

#### Gastric cancer (GC)

**Figure 4:**
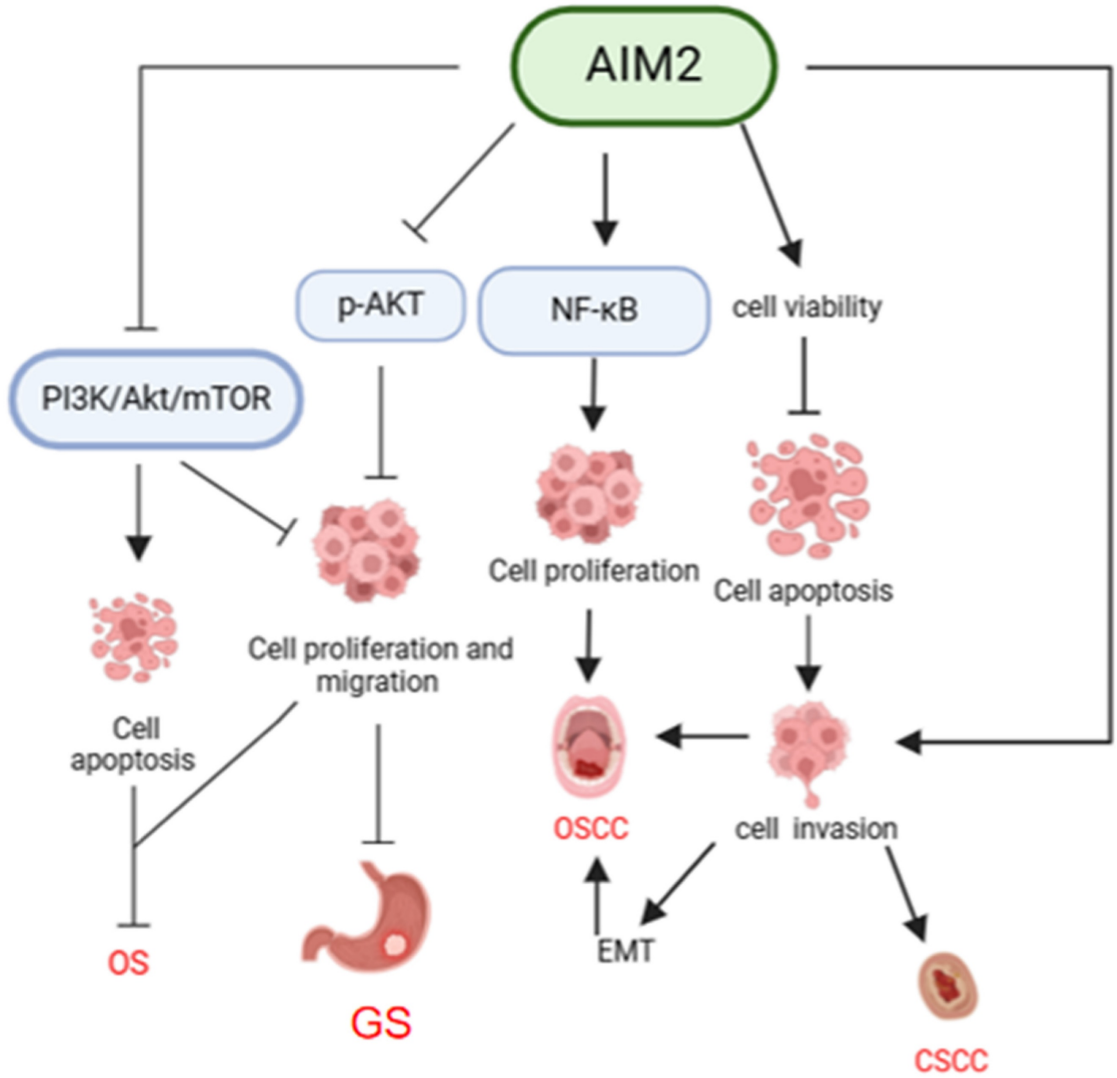
** Mechanisms of AIM2 regulation in OSCC, CSCC, GS, and OS independent of inflammasome.** AIM2 has been shown to play a role in promoting cancer cell proliferation through activation of the NF-κB signaling pathway, as well as enhancing cancer cell viability and inhibiting apoptosis. This ultimately leads to the promotion of cancer cell invasion and the development of EMT, thereby contributing to the progression of OSCC and CSCC. Conversely, in GS, AIM2 has been found to inhibit cell growth and migration by suppressing AKT phosphorylation. Additionally, in OS, AIM2 can inactivate the PI3K/AKT/mTOR pathway, leading to inhibition of proliferation, invasion, and migration of osteosarcoma cells, as well as promotion of apoptosis, ultimately hindering the progression of OS.

GC is recognized as a significant malignancy globally, characterized by a substantial disease burden and high mortality rates^67^, ^68^. Research has revealed a notable decrease in AIM2 expression within GC tumor tissues, with AIM2 deficiency correlating with factors such as tumor size, lymph node metastasis (LNM), tumor, lymph node metastasis (TNM) stage, and unfavorable prognostic outcomes in GC patients. Experimental findings further indicate that knockdown of AIM2 in GC cells promotes cell proliferation and migration through the activation of AKT phosphorylation, while conversely, overexpression of AIM2 results in the opposite cellular phenotype^69^ (**Table 2, Figure 4**).

#### Osteosarcoma (OS)

OS is the predominant primary bone malignancy characterized by a significant tendency for local infiltration and metastasis. Despite advancements in treatment modalities such as surgical intervention and chemotherapy, the prognosis for patients with metastatic or recurrent osteosarcoma remains suboptimal^70^. AIM2 expression is notably diminished in diverse OS cell lines, and upregulation of AIM2 has been shown to impede the PI3K/AKT/mTOR signaling pathway, thereby suppressing cellular proliferation, invasion, migration, and EMT while promoting apoptosis. This inhibitory effect on OS progression is reversed upon AIM2 knockdown^19^ (**Table 2, Figure 4**).

### The potential benefits and associated challenges of AIM2 in cancer therapy

AIM2 functions as a significant regulatory factor in the context of cancer. Its roles appear to vary across different types of cancers, distinct tumor microenvironments, and various stages of cancer progression, potentially operating through both inflammasome-dependent and inflammasome- independent mechanisms.

**Figure 5:**
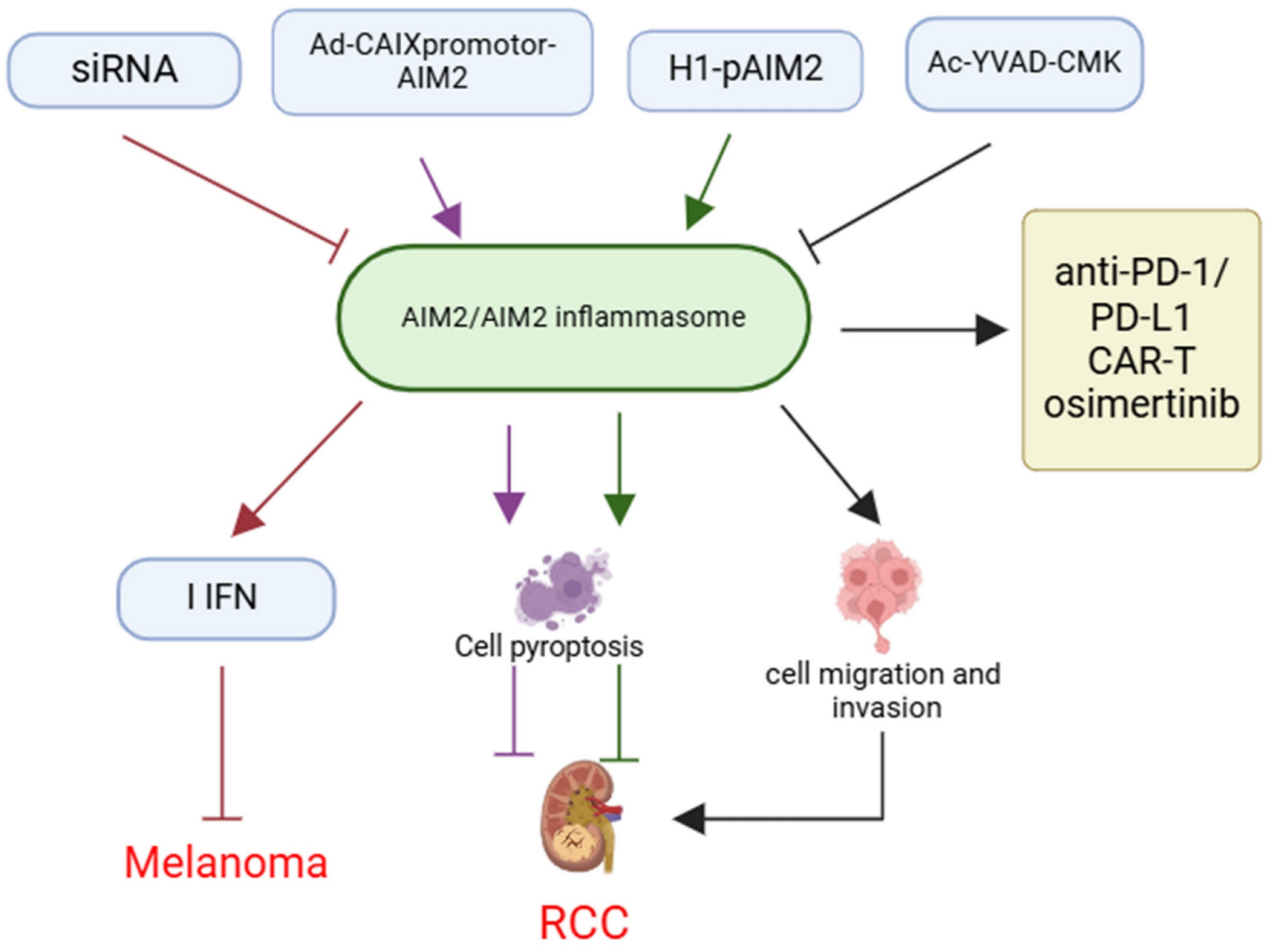
** The implication and application of AIM2 in cancer therapy.** Therapeutic strategies targeting AIM2, such as siRNA, Ad-CAIXpromotor-AIM2, H1-pAIM2, and Ac-YVAD-CMK, inhibit melanoma progression by modulating AIM2 or inflammasome activity and enhancing IFN expression. These approaches can also influence cancer cell pyroptosis, migration, and invasion, affecting RCC development. Combining AIM2-targeted therapies with existing immunotherapies like anti-PD-1/PD-L1 blockade, CAR-T cell therapy, or drugs like osimertinib may enhance treatment efficacy.

AIM2 has been demonstrated significant potential as a therapeutic target for tumor treatment, particularly in “cold tumors” where conventional immunotherapy proves ineffective. Research indicates that AIM2 expression in human melanoma DCs is correlated with poor prognosis and exhibits immunosuppressive properties. Furthermore, the inoculation of AIM2-deficient DCs may enhance the efficacy of relay T-cell therapy and anti-PD-1 immunotherapy in “cold tumors”^71^ (**Figure 5**).

Among the therapeutic strategies targeting AIM2, small interfering RNAs (siRNAs)^71^, inflammasome inhibitors^16^, conditional replication adenoviruses (CRAds)^21^, and gene delivery systems^72^ have demonstrated efficacy, particularly in preclinical animal models and *in vitro* experiments. These approaches significantly increase the sensitivity of tumor cells to the immune system by inhibiting the activity of AIM2 or inflammasomes, or by promoting the oncolytic effects of AIM2, thereby augmenting the anti-tumor immune response (**Figure 5**).

Notably, the integration of AIM2-targeted therapy with existing immunotherapeutic modalities, including anti-PD-1/PD-L1 inhibitors, chimeric antigen receptor T (CAR-T) cell therapy, and pharmacological agents such as osimertinib, holds potential for enhancing therapeutic outcomes, particularly in tumor types characterized by comparatively weak immune responses. Preclinical studies have demonstrated that these combination therapies can significantly improve patient response rates to treatment^73^-^75^ (**Figure 5**).

Despite the promising potential of AIM2-targeted therapies in the treatment of tumors, numerous challenges and limitations persist. First, AIM2-targeted therapy necessitates exceptionally high specificity to prevent unintended modulation of AIM2 activity in non-tumor tissues. Consequently, minimizing off-target effects while preserving therapeutic efficacy represents a significant challenge. Second, the expression levels and mechanisms of AIM2 action can vary substantially across different tumor types and individual patients. For instance, as previously discussed, AIM2 may exhibit divergent or even opposing effects in certain tumors due to distinct underlying mechanisms. This variability may influence the efficacy of targeted therapies, particularly within the realm of precision medicine, where tailoring therapeutic strategies to individual patient contexts presents a significant challenge. Furthermore, despite the promising results of AIM2-targeted therapies in preclinical studies, numerous obstacles remain in translating these findings into safe and effective clinical treatments. Notably, there is a scarcity of clinical-grade inhibitors targeting AIM2, and the design of clinical trials poses additional challenges.

In conclusion, the regulatory mechanism of AIM2 in cancer development and progression holds significant potential as a therapeutic target for the treatment of various cancers. However, considerable challenges remain to be addressed.

## Discussion

The intricate tumor microenvironment is a critical factor in the promotion of tumor growth. AIM2, a dsDNA sensor from the interferon-inducible p200 family, is believed to have a dual function in both innate immune response and tumor pathogenesis^76^, ^77^. AIM2 is significantly involved in the initiation and advancement of numerous types of cancer, although its expression and mechanisms vary among different cancer types.

Prior research has primarily concentrated on the involvement of AIM2 in different types of cancer, particularly in relation to inflammasome signaling. The inflammasome serves a crucial defensive function within the human body, with the AIM2 inflammasome exhibiting antibacterial properties and the ability to inhibit certain bacterial pathogens^78^. Furthermore, activation of the inflammasome is essential for safeguarding against viral infections, while AIM2 inflammasome also has the capacity to identify and protect against fungal pathogens^4^, ^79^, ^80^. The inflammasome is capable of recognizing a diverse array of pathogen-associated molecules, leading to its activation by DNA and RNA viruses and subsequent induction of pyroptosis^78^, ^81^. While the primary function of the inflammasome is to provide defense against pathogens, prolonged and excessive inflammation can have detrimental effects^81^. Chronic inflammation has been implicated in various stages of tumorigenesis, impacting processes such as tumor initiation, promotion, malignant transformation, invasion, and metastasis. The growth of initial tumors may be influenced by inflammation-driven stimulation. The tumorigenic properties of inflammatory mediators encompass a variety of mechanisms, such as augmented cellular proliferation, inhibition of apoptosis, immune suppression, facilitation of tumor cell migration and invasion, and promotion of angiogenesis^76^, ^82^, ^83^.

This review highlights AIM2 as the initial non-NLR family inflammasome implicated in various cancer types, underscoring its significant involvement in tumorigenesis irrespective of inflammasome activation.

From a perspective of tumor promotion and suppression, AIM2 exhibits diverse roles across different types of cancers. Studies have shown a decrease in AIM2 expression in specific cancer types including CRC, breast cancer, HCC, HSCC, GC, and OS. The reduced levels of AIM2 suggest a potential tumor suppressor function in these cancers. AIM2 has been shown to impede the malignant behaviors of CRC cells, such as proliferation and migration, through modulation of the AKT/mTOR signaling pathway, NF-κB signaling pathway, Notch signaling pathway, mitochondrial dynamics, and cell cycle regulation, thereby exhibiting anti-tumor effects. Furthermore, the upregulation of AIM2 has been observed in various cancers, including lung cancer, RCC, OSCC, and CSCC, where it has been shown to enhance cell viability, invasiveness, and suppress apoptosis primarily via the JAK/STAT3 pathway, inhibition of iron death, and the NF-κB signaling pathway, ultimately promoting tumorigenesis. Conversely, AIM2 may exhibit distinct functions in HCC and cervical cancer, potentially through unique mechanisms.

AIM2 plays a multifaceted role in tumorigenesis, demonstrating both promotional and inhibitory effects on cancer development. Its functions include promoting cell proliferation, migration, and invasion, as well as suppressing anti-tumor immune responses.

The role of AIM2 in inflammation and tumor response is not singular; rather, it involves interactions with various molecules, forming a complex regulatory network. Specifically, molecules intimately associated with AIM2 function, including Interferon-inducible protein 16 (IFI16), ASC, Caspase-1, IL-18, and IL-1β, contribute distinct roles in the assembly and signaling of inflammasomes. Recent research has elucidated the diverse functions of these molecules across different tumor types.

The function of IFI16 in various cancers exhibits a dual nature. In BC and triple-negative breast cancer (TNBC), IFI16 is implicated in the inhibition of tumor growth and migration^84^, ^85^. Conversely, elevated levels of IFI16 in the serum of BC patients are associated with pro-tumor inflammation and disease progression^86^. Additionally, in esophageal squamous cell carcinoma (ESCC), IFI16 facilitates the development of a malignant phenotype and is significantly correlated with the invasive characteristics of the tumor^87^. These observations suggest that IFI16 may operate through distinct mechanisms across different cancer types. Similar to the dual role of IFI16, ASC facilitates the progression of GC and pancreatic ductal carcinoma by enhancing the expression of IL-18 or IL-1β, which subsequently promotes the development of pancreatic ductal carcinoma^88^, ^89^. Conversely, in CRC, ASC is implicated in the development of carcinoma through the production of mitochondrial ROS and the activation of JNK signaling, leading to cell necrosis and exerting tumor-suppressive effects^90^. Caspase-1 can inhibit the development of BC and CRC by promoting cellular pyroptosis^91^, ^92^. In contrast, IL-1β has been extensively studied as a pro-tumor factor, exhibiting oncogenic effects in various cancers. The absence of IL-1β has been shown to inhibit the development of fibrosarcoma in murine models^93^. Conversely, in prostate cancer, pancreatic cancer, and HCC, IL-1β has been found to accelerate tumor progression by inducing immunosuppression and promoting inflammation and metastasis^94^-^96^. As for IL-18, it primarily exhibits pro-tumor properties across various cancer types, facilitating tumor progression and metastasis through mechanisms such as enhanced angiogenesis, promotion of cell migration, and immune evasion^97^-^101^. The multifaceted roles of these molecules in distinct tumor contexts imply that their functions may be contingent upon specific tumor types and their microenvironments. Consequently, further elucidation of these mechanisms is imperative for the advancement of novel therapeutic strategies for cancer treatment.

In summary, AIM2 functions as a critical regulator in cancer pathogenesis, demonstrating a range of roles across different cancer types, tumor microenvironments, and stages of disease progression, both in inflammasome-dependent and inflammasome-independent contexts. Consequently, understanding the mechanisms governing AIM2 regulation in cancer development and progression represents a promising therapeutic strategy for various malignancies, albeit with significant challenges. Therefore, the advancement of versatile, effective, and precise molecularly targeted cancer therapies is imperative and should be prioritized.

## Figures and Tables

**Table 1:** Role of AIM2 in cancer development: independence on the inflammasome.

Diseases	Cells	Research Methods	Mechanism	Function	Reference
RCC	786-O, OSRC-2	*in vivo* and *in vitro* experiments.	AIM2 conditionally replicating adenovirus (Ad-CAIX^promotor^ -AIM2) could inhibit cell proliferation, promote apoptosis and killing of cancer cells through the inflammasome, induce tumor lysis, and also inhibit lung metastasis in mice with renal cancer.	Inhibition	21
	BMDMs, Renca cells	*in vivo* and *in vitro* experiments.	AIM2 enhanced the polarization switch of tumor-associated macrophages (TAMs) from anti-inflammatory M2-type to pro-inflammatory M1-type through the activation of inflammasome signaling, which promoted tumor rejection and thus inhibits tumor cell growth.	Inhibition	16
HCC	MHCC97H, MHCC97L, BEL7402, SMCC7721, HepG2	Clinical research; *in vivo* experiments.	AIM2 was found to inhibit the mTOR-S6K1 pathway by inducing cellular pyroptosis through the formation of inflammasomes and inhibiting the proliferation, colony formation and invasion of HCC cells.	Inhibition	22
	Kupffer cell	*in vivo* and *in vitro* experiments.	AIM2 promoted the activation of hepatic inflammasomes and participated in the early inflammatory and proliferative responses in HCC.	Promotion	23
Cervical cancer	SiHa, ME-180, CaSki, SNU-17, HeLa	*in vivo* and *in vitro* experiments.	HPV-infected cervical cancer cells could continue to grow by inhibiting AIM2 inflammasome-mediated immunity through SIRT1.	Inhibition	24
Nasopharyngeal carcinoma	NPC-TW01, -TW02, -TW04, HK1	*in vivo* and *in vitro* experiments.	The activation of AIM2 inflammasome promoted the secretion of IL-1β and recruited tumor-associated neutrophils (TANs), thereby inhibiting tumor growth in mice.	Inhibition	26
Prostate Cancer	Human normal prostate epithelial cells (PrECs), THP-1	*In vivo* experiment.	AIM2 inflammasomes promoted chronic inflammation in the prostate gland, thereby promoting prostate cancer.	Promotion	27
LUAD	A549, H1355, HCC827, PC9	*In vivo* experiment.	AIM2 mRNA expression levels were upregulated in lung cancer tissues compared to normal tissues and positively correlated with poor prognosis. The overexpression of AIM2 promoted the transcriptional regulation of NF-κB and STAT1 by enhancing the expression of IL-1β and IL-18, which in turn promoted the EMT process and PD-L1 expression.	Promotion	18
	A549, H460	*in vivo* and *in vitro* experiments.	AIM2 short hairpin RNA (shRNA)-mediated inhibition of cell proliferation was triggered by cell accumulation in G2/M phase. The overexpression of AIM2 induced inflammasome formation, resulting in increased levels of cleaved caspase-1 and mature IL-1β in NSCLC cell lines, which in turn enhanced cell viability and migration of NSCLC cell lines.	Promotion	29
	Plasmacytoid dendritic cells (pDCs)	Clinical research; *in vivo* experiments.	Tumor-associated pDCs responded to AIM2 activation and promoted calcium efflux and reactive oxygen species from the mitochondria, leading to calpain activation and high levels of IL-1α, which promoted lung tumor cell proliferation.	Promotion	30

**Table 2:** Role of AIM2 in cancer development: dependence on the inflammasome.

Diseases	Cells	Research Methods	Mechanism	Function	Reference
CRC	HCT116, 293T, HT-29	Microarray analysis; *in vitro* experiments.	AIM2 triggered the induction of HLA-DRA and HLA-DRB by modulating CIITA expression, and finally acted through the IFN / AIM2 / ISG cascade to function in CRC.	Inhibition	37
	HCT116	*in vivo* experiment.	AIM2 inhibited CRC cell viability and increases apoptosis by suppressing the PI3K/AKT pathway to exert antitumor effects.	Inhibition	38
	HCT116	*in vivo* experiment.	AIM2 delayed the cell cycle transition from G2 to M phase, thereby slowing proliferation and inhibiting the growth of colon cancer cell lines, and suppressing invasion of CRC cells, but did not induce apoptosis.	Inhibition	39
	SW480, SW620, HCT116, LoVo	*in vivo* and *in vitro* experiments.	AIM2 inhibited the proliferation and migration of CRC cells by regulating AKT/mTOR and signaling pathway, and inhibiting the expression of Glil.	Inhibition	17
	BMDMs, MEFs, HCT116, HEK293T	*in vivo* and *in vitro* experiments.	AIM2 inhibited colon tumorigenesis and progression by limiting DNA-PK and Akt activation.	Inhibition	40
	Intestinal stem cells	*in vivo* experiment.	AIM2 inhibits the development of CRC by suppressing enterocyte proliferation, expansion of intestinal stem cells, and regulation of the microbiota.	Inhibition	41
Breast cancer	MCF-7, Tet-Off	*in vivo* experiment.	AIM2 promoted apoptosis in breast cancer cells through a mitochondrial mechanism, and also inhibited the expression of the anti-apoptotic protein Bcl-xL, increased the expression of the apoptotic proteins Bad and Bax, and activated cysteine asparaginase, leading to cleavage of the DNA repair protein PARP.	Inhibition	44
	MCF-7, MDA-MB-231	*in vivo* and *in vitro* experiments.	AIM2 promoted the expression of Caspase 3 and DFNA5 to induce breast cancer cell death, thereby suppressing tumorigenesis.	Inhibition	45
RCC	786-O, OSRC-2	*in vivo* and *in vitro* experiments.	Overexpression of AIM2 inhibited cell proliferation, migration and invasion, and enhanced autophagy in renal cancer cells, thereby inhibiting the malignant biological behavior of renal cancer cells.	Inhibition	47
	786O, OSRC2, Caki-1, A498, ACHN	*in vivo* and *in vitro* experiments.	AIM2 promoted phosphorylation and proteasomal degradation of FOXO3a, which reduced its transcriptional effect on ACSL4, inhibited iron death, and promoted renal carcinoma development.	Promotion	50
HCC	Bel-7402, SMMC-7721, Huh7, Be-7404	*in vivo* and *in vitro* experiments.	Low expression of AIM2 was strongly associated with higher serum AFP levels, vascular infiltration, poor tumor differentiation, incomplete tumor envelope, and lower postoperative survival. Deletion of AIM2 promoted EMT activation and HCC metastasis.	Inhibition	53
	HCCLM3, SMMC-7721	Clinical research; *in vivo* and *in vitro* experiments.	AIM2 promoted apoptosis and inhibited the migration and invasion of cancer cells by inhibiting the Notch signaling pathway, and delayed tumor progression in homograft experiments using nude mice, thereby inhibiting HCC growth and metastasis.	Inhibition	54
LUAD	Raw264.7 cells, LA795 cells	Clinical bioinformatic analysis; *in vivo* and *in vitro* experiments.	AIM2 promoted immune escape from LUAD by inducing M2 polarization of macrophages and PD-L1 expression via the JAK/STAT3 pathway and by inhibiting CD8^+^ T cell infiltration through the PD-1/PD-L1 axis.	Promotion	57
	H1975, H358, A549, H157, HCC827, H3255, H460	*in vivo* and *in vitro* experiments.	AIM2 promoted cancer cell proliferation by regulating mitochondrial dynamics, resulting in decreased mitochondrial fusion, which in turn led to increased cellular reactive oxygen species production and activation of the MAPK/ERK signaling pathway.	Promotion	58
SCC	UT-SCC12A, -91, -105, -111 and -118; UT-SCC7, -59A and -115	*in vivo* and *in vitro* experiments.	Down-regulation of AIM2 expression decreased cell viability in CSCC cells, triggered apoptosis, resulted in decreased cell invasion, and inhibit growth and vascularization of CSCC xenografts *in vivo*.	Promotion	62
	Ca9-22, Ho1u1, HSC2, HSC3, HSC4, HSQ89, SAS and Sa3	*in vivo* experiment.	When p53 is absent, the co-expression of AIM2 and IFI16 promoted cell proliferation by activating the NF-ĸB signaling pathway. Meanwhile, caspase-1 was not activated in oral squamous cell carcinoma cells. dsDNA could not trigger the formation of AIM2 inflammasome in OSCC cells.	Promotion	63
	HSC2, HSC3, HSC4, HSQ89, Ho-1-u-1, SAS, Sa3 and Ca922	*in vivo* and *in vitro* experiments.	AIM2 overexpression contributed to the tumorigenesis of OSCC and caused high migration of cancer cells, increased the invasive ability of cancer cells, and led to enhanced EMT. Moreover, *in vivo* experiments by *in situ* transfer into immunodeficient mice also showed that AIM2-overexpressing cancer cells resulted in enhanced tumor growth in the tongue and reduced survival in mice.	Promotion	65
	HSC2, HSC3, HSC4, SAS	Clinical bioinformatic analysis; *in vitro* experiments.	AIM2 promoted radiation resistance, migration and PD-L1 expression in oral squamous carcinoma cells through activation of STAT1/NF-κB.	Promotion	64
	/	Clinical bioinformatic analysis and clinical sample research.	Low AIM2 expression combined with high p-STAT3 expression is closely related to lymph node metastasis, intravascular tumor thrombosis, low survival rate and poor prognosis.	Inhibition	66
GC	MGC803, SGC7901, MKN45, AGS	Clinical research; *in vitro* experiments.	AIM2 inhibited GC cell proliferation and migration by suppressing AKT signaling.	Inhibition	69
OS	hFOB1.19, C396, CAL-72, MG-63	*in vivo* experiments.	AIM2 inhibited proliferation, invasion and migration and promoted apoptosis of osteosarcoma cells by inactivating the PI3K/AKT/mTOR signaling pathway.	Inhibition	19
